# Redirection of Care after Traumatic Brain Injury in Intensive Care: Sex and Social Determinants of Health

**DOI:** 10.1177/08977151251360617

**Published:** 2025-07-22

**Authors:** Simone Unseld, Alessandra Nadja Herzog, Federica Stretti, Tanja Krones, Caroline Hertler, Giovanna Brandi, Stefan Yu Bögli

**Affiliations:** ^1^ Institute of Intensive Care Medicine, University Hospital of Zurich, University of Zurich, Zurich, Switzerland.; ^2^ Institute of Biomedical Ethics and History of Medicine, Clinical Ethics, University Hospital of Zurich, University of Zurich, Zurich, Switzerland.; ^3^ Department of Radiation Oncology and Competence Center for Palliative Care, University Hospital of Zurich, University of Zurich, Zurich, Switzerland.; ^4^ Department of Neurology, Clinical Neuroscience Centre, University Hospital of Zurich, University of Zurich, Zurich, Switzerland.

**Keywords:** end of life, sex differences, social determinants of health, traumatic brain injury

## Abstract

Traumatic brain injury (TBI) impairs a patient’s capacity for informed decision-making, necessitating surrogate decision-makers to decide whether to continue life-sustaining therapies. Patient sex and social determinants of health (SDH)—for example, economic stability, education, and health care access—possibly affect such decisions. We aimed to explore interactions between sex, SDH, and redirection of care in a cohort of patients with TBI from a high-income, high-resource country. Adult patients with consecutive TBI admitted to intensive care were included. Data on demographics, TBI characteristics, advance directives, and SDH (civil status, living situation, dependence for daily activities, income, employment, religion, nationality) were extracted. The primary end-point was redirection of care, followed by in-hospital mortality. Differences were analyzed univariably, after prognostic score matching, and through random forest models to assess the importance of each factor. Seven hundred and twelve patients (26.4% female, median age 56) were included. Women were older, more often widowed, and more frequently dependent on help, while men had higher income and education levels. Redirection of care and mortality were more common in women, even after prognostic score matching, though the difference disappeared after adjusting for redirection of care. Random forest models identified employment status and dependence on support as key factors associated with redirection of care, while sex did not improve model performance. Our results underline the importance of SDH for prognostication of patients with TBI and suggest that it is not sex *per se*, but the associated sex differences in SDH that affect the frequency of redirection of care and ultimately in-hospital mortality.

## Introduction

Traumatic brain injury (TBI) remains an important cause of disability and death across the world.^1–4^ Long-term outcomes vary widely between patients, even in the most severe cases.^5^ Often, surrogate decision-makers (SDMs) are faced with the question of whether to continue full treatment, to restrict/withhold further interventions, or even to withdraw life-sustaining therapies.^6^ Whether redirection of care is considered is often complicated by missing advance directives (ADs) and patients’ altered brain function rendering them unable to participate in the end-of-life (EOL) discussions. There is a lack of reliable prognostic models, leading to substantial barriers in recommending and deciding whether to withdraw life-sustaining treatment in patients with severe TBI.^7^ Importantly, the outcome is not only determined by clinical features of the disease (such as the patients’ age or TBI descriptors such as the Glasgow Coma Scale [GCS] or the Injury Severity Scale [ISS]) but also critically influenced by complex cultural and regional factors.^8,9^

In recent years, so-called social determinants of health (SDH) are considered for improving prognostication. SDH are defined as nonmedical factors that influence health outcomes,^10,11^ affecting the patients’ disease trajectory. These can include level of education, economic stability (e.g., level of income and employment status), or availability of health care. Additionally, more complex factors represent important SDH, such as the living situation or even the patients’ race or nationality.^12,13^ Differences in these factors lead to health disparities and consequently different outcomes in spite of similar disease severity. We previously demonstrated that women admitted to neurocritical care received withdrawal of life-sustaining therapies more often in spite of similar disease severity.^14^ Based on these findings and previous explorations concerning SDH, the current analysis had the following aims: (1) to assess whether sex differences in SDH exist in our cohort of patients from a high-income, high-resource country and (2) to assess whether these SDH can explain part of the currently unexplained difference in the frequency of redirection of care between sexes after TBI.

## Materials and Methods

This single-center, retrospective analysis of an observational TBI registry was conducted at the Institute for Intensive Care, University Hospital of Zurich in Switzerland. The local ethics committee (Kantonale Ethikkommission Zürich) approved the study. It was performed in accordance with the ethical standards as laid down in the Declaration of Helsinki. Informed consent was waived for the development of the database (including the acquisition of routinely available information). Patients were prospectively screened for eligibility and included if general consent for the extraction of data was available. All adult patients (≥18 years of age) with TBI admitted to the intensive care unit (ICU) of the University Hospital Zurich between January 2018 and December 2022 were screened for eligibility. Patients were excluded if the time interval between trauma and hospital admission was greater than 24 h, or if they refused analysis of their data (documented written or oral refusal). The data were obtained from the electronic medical records. Study data were collected and managed using Research Electronic Data Capture (REDCap, Nashville, USA).

### Extracted data

Collected demographic data include age, sex, and comorbidities based on the Charlson Comorbidity Index (CCI).^15^ The severity of the injury was assessed using frequency of isolated TBI (extracranial Abbreviated Injury Scale [AIS]^16^ <3), the ISS,^17^ as well as initial GCS^18,19^ and the core IMPACT score (IMPACTcore).^20^ Additionally, pupillary reactivity, prehospital hypotension and hypoxia, and initial blood glucose and hemoglobin values were assessed.

The following SDH were extracted from the electronic patient files: civil status (divorced/separated vs. partnership/marriage vs. single vs. widowed), living situation (alone at home vs. with other persons at home vs. retirement/nursing home), dependence on support for the activities of daily life, income category (high vs. middle vs. low), type of insurance (public vs. private), level of education (basic [9 years] vs. higher [12 years] vs. university education), employment status (employed/working/in training vs. retired vs. unemployed), religion (Christian vs. religiously unaffiliated vs. other), nationality (Swiss vs. other), spoken language (German vs. other), and place of residence (urban vs. rural, i.e., above vs. below 5000 inhabitants). In addition to the patient-specific information, additional information was extracted from the place of residence (i.e., municipality) allowing for capturing regional differences. The canton of Zurich is made up of 160 distinct municipalities with a median of 4674 (interquartile range [IQR]: 1938–7966) inhabitants. The information was extracted from the Federal Statistical Office in Zurich, who periodically receive the information from the municipalities (the last full description was performed at the end of the year 2021). This additional information was only available for patients living in Zurich; for those outside Zurich, median imputer was employed. This method allowed us to capture the following additional regional information: welfare dependence (%), unemployment rate (%), median income (per inhabitant), median assets (per inhabitant), and density of private practice physicians (per 1000 inhabitants).

Extracted outcome metrics were ICU length of stay (LOS) and in-hospital mortality. Redirection of care was explored by assessing frequency, type (palliation vs. limited [existing life-support therapy is not intensified further]), timing (early—i.e., within 24 h vs. late—i.e., thereafter), and reason (medical reason [the Swiss law allows for redirection of care based on medical reason if death or inacceptable quality of life is inevitable] vs. known or presumed patient’s will). ADs (which are filed electronically in the patient’s records) were analyzed, assessing whether the patient preferred palliative measures in case of loss of capacity. In case no written AD was available, the patient’s presumed wishes were evaluated based on the assessment by the SDM.

### Statistical analysis

Statistical analysis and figure preparation were performed in R Studio (R version 4.3.2—www.r-project.org/—packages used: *tidyverse*, *gtsummary*, *MatchIt*, *optmatch*, *cobalt*, *survminer*, *survival*, *randomForest*, *caret*, *ggplot2*). Descriptive statistics are reported as counts/percentages or as median including the IQR as appropriate. Categorical variables are compared with Pearson’s χ^2^ or Fisher’s exact test, continuous/ordinal variables using the Wilcoxon rank-sum test. To assess possible interactions between the different characteristics and outcome, multivariable Cox proportional hazard regression and logistic regression analyses were performed.

### Prognostic risk score matching and random forest trees

Prognostic risk score matching was performed to adjust for the differences in TBI characteristics and age between sexes. The covariates considered for matching were: age, CCI, GCS M (GCS motor score), pupillary reactivity, ISS, isolated TBI versus polytrauma, lowest preadmission incidence of hypotension and hypoxia, and blood glucose. Median imputer was used for missing data within these covariates (the amount of missing data was: 6% for blood glucose and pupillary reactivity, 3% for hemoglobin, and 5% for initial GCS M). The differences in AD and SDH descriptors were then tested both univariably. Considering the remaining differences in frequency of redirection of care, a second prognostic risk score matching was performed including the frequency of redirection of care in addition to the above-mentioned clinical descriptors.

Lastly, we employed a random forest classification approach to examine the influence of the various SDH and sex on the incidence of redirection of care. In addition to the SDH and sex, age and stated preference for EOL (within available ADs) were added to the models. The level of education was excluded since it was missing in over 30% of matched patients. Additionally, we excluded neighborhood-derived metrics, median income, and proportion of unemployment, since these displayed strong collinearity with median assets and welfare dependence, respectively. For each iteration, the control dataset was randomly divided into training (70%) and testing (30%) sets. To address potential class imbalance, the random forest was configured with appropriate class weighting. One hundred decision trees (after tuning) were built to assess variable importance. Model performance was evaluated by comparing predicted labels with actual labels, yielding an area under the receiver operating characteristic curve to assess the model’s discrimination. Finally, variable importance outputs were reviewed to determine the relative impact of each social determinant on the likelihood of redirection of care.

### Data availability

The preprocessed data are available upon reasonable request by the corresponding author.

## Results

### Patient characteristics

Seven hundred and twelve patients with consecutive TBI were admitted to the trauma ICU between January 2018 and December 2022 (median age 56 [IQR: 36–74], 26.4% female—*n* = 188). Women were 10 years older than men (median 64 [IQR: 47–78] vs. 54 [IQR: 34–72] for women and men, respectively, *p* < 0.001). The TBI characteristics are described in Table 1. Seventy-three percent were affected by major trauma (ISS: >15), and 51% had sustained an isolated TBI. Median GCS was 13 (IQR: 7–14), and bilateral pupillary reactivity was preserved in 85%. However, women displayed a higher severity of cranial injury (*p* = 0.006). Furthermore, women more frequently had nonreacting pupils (18% vs. 10% for women and men, respectively, *p* = 0.034), and a higher IMPACTcore score (median 5 [IQR: 4–8] vs. 4 [IQR: 2–6] for women and men, respectively, *p* < 0.001). The severity of comorbidities was similar between sexes (median CCI of 0 for either sex, *p* = 0.8).

**Table 1.** Patient Characteristics

Characteristic	Overall *N* = 712	Female *N* = 188	Male *N* = 524	*p*-Value
GCS	13 (7–14)	13 (6–14)	13 (7–15)	0.12
GCS ≤ 8	220 (32%)	71 (39%)	149 (29%)	0.012
GCS motor score	6 (4–6)	5 (3–6)	6 (4–6)	0.11
ISS	22 (14–29)	25 (16–29)	22 (14–29)	0.14
ISS ≥ 25	318 (45%)	97 (52%)	221 (42%)	0.026
AIS head	3 (2–4)	3 (3–5)	3 (2–4)	0.006
Isolated TBI	364 (51%)	107 (57%)	257 (49%)	0.06
IMPACTcore	5 (2–7)	5 (4–8)	4 (2–6)	<0.001
Pupillary reactivity				0.034
Both reacting	566 (85%)	139 (79%)	427 (87%)	
One reacting	19 (2.8%)	6 (3.4%)	13 (2.6%)	
None reacting	82 (12%)	31 (18%)	51 (10%)	
Hypotension	87 (12%)	25 (13%)	62 (12%)	0.4
Hypoxia	224 (31%)	61 (32%)	163 (31%)	0.3
Glucose (mmol/L)	7.3 (6.1–8.9)	7.6 (6.2–9.0)	7.2 (6.0–8.9)	0.4
Hemoglobin (g/dL)	13 (11–14)	12 (10–13)	13 (12–15)	<0.001
Charlson Comorbidity Index	0 (0–1)	0 (0–1)	0 (0–1)	0.8

AIS, abbreviated injury scale; GCS, Glasgow Coma Scale; ISS, injury severity score; TBI, traumatic brain injury.

### Outcome

**FIG. 1.**
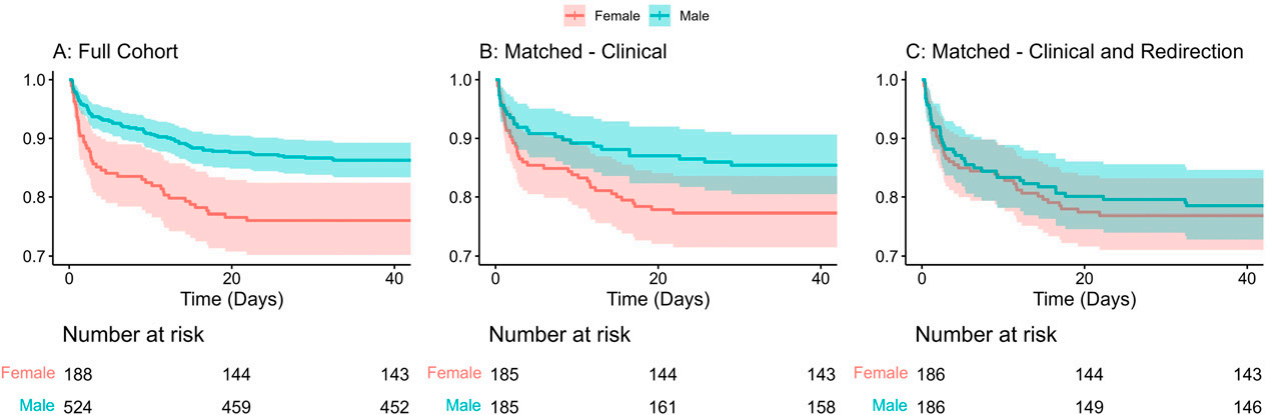
Kaplan–Meier curves comparing in-hospital mortality between sexes. **(A)** The overall mortality comparison; **(B)** the comparison after prognostic risk score-based matching that included the relevant clinical variables; and **(C)** the comparison after prognostic risk score matching for both clinical variables and incidence of redirection of care. A Cox proportional hazards model was conducted to evaluate differences in survival across the groups. The test revealed a statistically significant difference in the overall comparison (HR = 0.54, CI: 0.37–0.78, *p* = 0.001), which remained statistically significant after matching for clinical variables (HR = 0.61, CI: 0.38–0.99, *p* = 0.048), but not after matching for both clinical variables and redirection of care (HR = 0.94, CI: 0.61–1.45, *p* = 0.79). CI, confidence interval; HR, hazard ratio.

Overall, median ICU LOS was the same in both sexes (2 days [IQR: 1–7]), but in-hospital mortality was higher in women (24% vs. 14% for women and men, respectively, *p* < 0.001; Table 2). The frequency of in-hospital mortality over time is shown in Figure 1A. Women were more likely to receive redirection of care (37% vs. 22% for women and men, respectively, *p* < 0.001). A Cox proportional hazards regression model showed that males had a significantly lower risk of in-hospital mortality compared with females (hazard ratio [HR] = 0.54, 95% confidence interval [CI]: 0.37–0.78, *p* = 0.001). Overall, only 13% of patients had an AD, most of whom were women (20% vs. 10% for women and men, respectively, *p* = 0.001).

**Table 2.** In-Hospital Outcome and Redirection of Care Characteristics

Characteristic	Overall *N* = 712	Female *N* = 188	Male *N* = 524	*p*-Value
Redirection of care (yes)	186 (26%)	69 (37%)	117 (22%)	<0.001
Redirection of care—type^a^				<0.001
* *Palliation	78 (42%)	28 (41%)	50 (43%)	
Limited in content or time	104 (56%)	39 (57%)	65 (56%)	
Redirection of care—timing^a^				0.068
Early	108 (58%)	46 (67%)	62 (53%)	
Late	78 (42%)	23 (33%)	55 (47%)	
Redirection of care—reason^a^				0.10
Medical reason	65 (35%)	19 (28%)	46 (39%)	
Patient/SDM will	121 (65%)	50 (72%)	71 (61%)	
Intensive care length of stay (days)	2 (1–7)	2 (1–7)	2 (1–7)	0.6
Advance directive (yes)	92 (13%)	37 (20%)	55 (10%)	0.001
Preference EOL (yes)	72 (10%)	33 (18%)	39 (7%)	<0.001
In-hospital mortality	119 (17%)	46 (24%)	73 (14%)	<0.001

^a^ Of patients receiving redirection of care.

EOL, end of life; SDM, surrogate decision-maker.

### Social determinants of health

The SDH are described in Tables 3 (patient-specific SDH) and 4 (small area variations in SDH). Women were more often widowed (15% vs. 3.4% for men and women, respectively, *p* < 0.001), while men were more often single (21% vs. 32% for men and women, respectively, *p* = 0.007). There were no differences in living situation, but more women were dependent on help for the activities of daily living (16% vs. 7% for women and men, respectively, *p* < 0.001). Men had a higher income category (3.8% vs. 11% for women and men, respectively, *p* = 0.009) and a higher level of education (*p* = 0.009). Overall, women were more likely retired (56% vs. 35% for women and men, respectively, *p* < 0.001). When considering measures describing regional disparities (Table 4), no, or only negligible, differences could be found.

**Table 3.** Social Determinants of Health and Prior Fitness

Characteristic	Overall *N* = 712	Female *N* = 188	Male *N* = 524	*p*-Value
Insurance (private)	64 (9.1%)	21 (11%)	43 (8.3%)	0.2
Civil status				<0.001
Divorced/separated	63 (9.2%)	20 (11%)	43 (8.6%)	
Partnership/marriage	379 (55%)	97 (53%)	282 (56%)	
Single	199 (29%)	39 (21%)	160 (32%)	
Widowed	44 (6.4%)	27 (15%)	17 (3.4%)	
Living situation				0.052
Alone at home	185 (28%)	54 (31%)	131 (27%)	
With other persons at home	421 (64%)	104 (60%)	317 (65%)	
Retirement/nursing home	24 (3.6%)	11 (6.3%)	13 (2.7%)	
Dependent on support for activities of daily living (yes)	65 (9%)	29 (16%)	36 (7%)	<0.001
Income category				0.009
High	59 (9.4%)	6 (3.8%)	53 (11%)	
Middle	463 (74%)	129 (82%)	334 (71%)	
Low	106 (17%)	23 (15%)	83 (18%)	
Education				0.009
Basic	279 (62%)	55 (62%)	224 (62%)	
Higher	124 (28%)	32 (36%)	92 (26%)	
University	45 (10%)	2 (2.2%)	43 (12%)	
Employment status				<0.001
Employed/working/in training	325 (49%)	62 (36%)	263 (54%)	
Retired	269 (40%)	99 (56%)	170 (35%)	
Unemployed	72 (11%)	15 (8.5%)	57 (12%)	
Religion				0.022
Christian	340 (48%)	106 (56%)	234 (45%)	
Religiously unaffiliated	206 (29%)	45 (24%)	161 (31%)	
Other	166 (23%)	37 (20%)	129 (25%)	
Nationality (Swiss)	572 (80%)	161 (86%)	411 (78%)	0.033
Language (German)	613 (86%)	167 (89%)	446 (85%)	0.2

**Table 4.** Social Determinants of Health: Small Area Variations

Characteristic	Overall *N* = 712	Female *N* = 188	Male *N* = 524	*p*-Value
Place of residence (urban)	507 (71%)	145 (77%)	362 (69%)	0.037
Proportion welfare dependency	2.0 (1.7–4.3)	2.2 (1.7–4.3)	1.9 (1.7–4.3)	0.14
Proportion unemployment	2.2 (1.9–2.4)	2.4 (1.9–2.4)	2.2 (1.9–2.4)	0.074
Median income (in 1000 swiss francs)	55.9 (54.2–56.9)	55.2 (54.2–56.9)	56.0 (54.2–56.9)	0.5
Median assets (in 1000 swiss francs)	77 (54–93)	70 (54–93)	81 (54–93)	0.5
Density of private practitioners (per 1000 residents)	1.6 (0.9–4.7)	1.8 (0.9–4.7)	1.5 (0.9–3.9)	0.15

### Prognostic risk score matching: Clinical factors versus redirection of care

We applied prognostic risk score matching to assess whether the found differences in outcome and frequency of redirection of care reflect true sex differences or are merely related to differences in TBI severity or age. Three hundred and seventy patients (185 males and 185 females, respectively) could be matched without residual clinical differences (Supplementary Data S1). Yet, women were still more likely to receive redirection of care (36% vs. 25% for women and men, respectively, *p* = 0.024) and were more likely to suffer in-hospital mortality (23% vs. 15% for women and men, respectively, *p* = 0.034). A Cox proportional hazards regression model showed that males had a significantly lower risk of in-hospital mortality compared with females even after correction for the clinical prognostic parameters (HR = 0.61, CI: 0.38–0.99, *p* = 0.048; Fig. 1B). There were also similar differences when comparing the differences in SDH (Supplementary Data S2). Considering the remaining differences in frequency of redirection of care, we performed a second prognostic risk score matching including the clinical descriptors (as above), but also adding the frequency of redirection of care to the variables used for prognostic risk score estimation. After matching, no difference in frequency of redirection (36% vs. 30% for women and men, respectively, *p* = 0.2) nor mortality (24% vs. 22% for women and men, respectively, *p* = 0.7) could be found. Additionally, no residual differences in the temporal trends of mortality could be identified (Fig. 1C).

### Random forest models

**FIG. 2.**
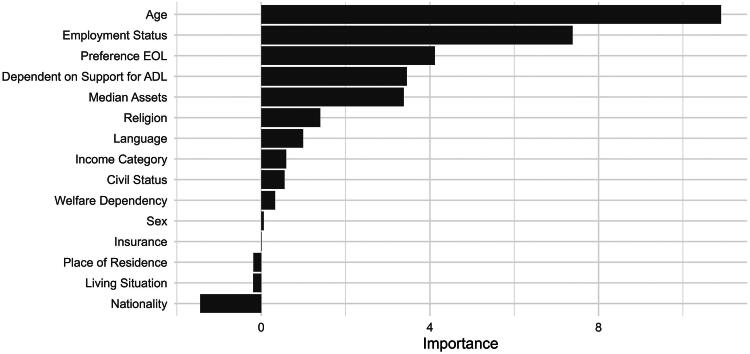
Random forest variable importance. This bar chart illustrates relative variable importance from a random forest model examining whether the frequency of redirection of care depends on various social determinants of health, as well as age and stated preference for EOL (both added due to the clear importance for such decisions). Higher scores along the *x*-axis indicate a greater predictive contribution to the model. Among the variables considered, age, employment status, and preference for EOL displayed the largest importance, followed by factors such as dependence on support for activities of daily living, median assets, and religion. EOL, end of life.

Because the findings suggest that redirection of care was the primary factor associated with in-hospital mortality after controlling for clinical parameters, we examined which factors influenced the frequency of redirection of care, with particular attention to potential biases related to SDH. We employed a random forest model to assess the relative importance of various SDH, as well as age and stated EOL preferences (both added due to their clear importance for such decisions), in predicting redirection of care. Age emerged as the most influential factor, followed by employment status, stated EOL preferences, and dependence on support for activities of daily living (Fig. 2). Contrary to earlier analyses, in comparison with the various SDH, sex did not affect the accuracy of the resulting models. The final model achieved an accuracy of 0.91, with a sensitivity of 0.82 and a specificity of 0.97.

## Discussion

Recent studies have highlighted various facets of SDH that affect the incidence of TBI, including the effects of education, income, and geographic factors.^10^ SDH are the conditions in which people are born, grow, live, work, and age, that impact their health outcomes. These determinants are shaped by the distribution of money, power, and resources at global, national, and local levels. We explored various SDH at the individual level (e.g., socioeconomic, education, nationality, and civil status) and neighborhood level (e.g., population, welfare dependency, and accessibility of medical care). Instead of focusing on these well-described associations with TBI incidence,^10^ we were interested in evaluating the effect on the postinjury management and outcomes of these patients. In pediatric TBI, such differences have long been identified, and the detrimental effect on access to rehabilitation services and outcome is well described.^21^ In adults, insurance status^22^ and socioeconomic deprivation^23^ have been shown to be associated with mortality after TBI. Additionally, previous research highlighted the importance of SDH in a large cohort of neurocritical care patients for the prediction of outcome after correction for the initial clinical status.^24^ Similarly, sex disparities have been recognized to influence outcomes.^25^ We identified sex differences in SDH even in the high-income, high-resource country of Switzerland. The included female patients had lower incomes, were less likely to reach the highest levels of education and were more likely to be dependent on help for activities of daily living. Overall, women admitted with TBI to the ICU suffered more severe injuries. Although higher TBI severity initially appeared to be the obvious culprit behind the mortality difference, the rate of care redirection emerged as the principal contributor to the increased mortality in women in our cohort, after we adjusted for the various clinical factors known to affect outcomes. Considering the known sex differences in SDH and the relevance of redirection of care for mortality, we explored the interaction between these factors using random forest trees, which allowed us to iteratively assess the importance of various predictors. Interestingly, SDH, rather than sex, emerged as the factor most closely associated with redirection of care. Overall, the results of this analysis suggest that differences in SDH mediate the effect of patient sex on outcome, rather than sex directly influencing outcome.

### Limitations

The main limitation arises from the single-center design with retrospective extraction of some of the information. Despite the moderate cohort size, all included patients were treated in a high-volume trauma ICU, which might have led to a bias in severity as well as treatment when comparing with smaller centers. Consequently, the various known regional differences in treatment regimens and EOL processes are not covered. Additionally, other known factors necessary for the prediction of in-hospital mortality, such as imaging-based descriptors of the severity of injury (except for AIS), information on the clinical course, and secondary worsening were not assessed. In addition, whether the SDH directly affect decision-making within the ICU or are themselves mediated through other factors cannot be determined within this analysis, since detailed information describing the decision-making process that led to the decision for redirection of care was missing. Previous studies elucidated the importance of health care access and insurance status on outcome, a result that we could not reproduce in our cohort, potentially due to the relatively high standard of living and dense health care system in Switzerland, limiting wider generalizability. Lastly, most patients were Swiss, German-speaking, and either Christian or religiously unaffiliated, making it a rather homogenous cohort in terms of background and potential racial bias. These aspects likely alter the decision process, since there are various regional and cultural differences that affect the presence and utilization of ADs and the availability or frequency of redirection of care.^8,26,27^

Of note, while assessing sex-related differences in the presence of ADs was not the primary aim of this analysis, it is important to note that only few, mostly female, patients admitted had an AD in our cohort, in contrast to a previous review^28^ and our cohort of neurocritically ill patients^14^ (which included patients with various neurological diseases including brain tumors). One potential explanation is the acute/sudden nature of TBI and the relatively young patient cohort, leading to the number being much closer to ones found within general ICU populations.^29,30^ While the presence of an AD is associated with earlier limitation of life-sustaining therapies, it does not *per se* increase the frequency of in-hospital mortality,^14,31^ and violations of the patient’s wishes might even negatively affect outcome.^29^ The scarcity of ADs found in this population might have allowed for more subtle influences such as SDH to alter management decisions, since the presence of ADs is strongly associated with decisions following their preferences.^32^

## Conclusions

The decision-making process following TBI remains poorly understood and is influenced by numerous nonclinical factors, including SDH, which may impact care decisions in unconscious and subtle ways. Our study highlights that sex-related differences in SDH are present and that SDH, rather than sex itself, are independently associated with the redirection of care, even after adjusting for disease severity, age, sex, and preferences stated in ADs. These findings suggest that SDH mediate the observed relationship between sex and care decisions. Moving forward, it is crucial to develop and implement clinical guidelines aimed at mitigating the influence of unconscious biases related to SDH in order to promote equitable decision-making in critical care.

## Data Availability

Postprocessed data are available upon reasonable request to the corresponding author.

## Abbreviations Used

AD: advance directives
AIS: Abbreviated Injury Scale
CCI: Charlson Comorbidity Index
CI: confidence interval
EOL: end of life
GCS: Glasgow Coma Scale
GCS M: GCS motor score
ICU: intensive care unit
ISS: injury severity score
OR: odds ratio
SDH: social determinants of health
TBI: traumatic brain injury
