# Supplementary material for: Redirection of Care after Traumatic Brain Injury in Intensive Care: Sex and Social Determinants of Health
Source: Neurotrauma Rep. 2025 Jul 22;6(1):569–77. doi: 10.1177/08977151251360617 (PMC12419443; doi:10.1177/08977151251360617)
Supplement: Supplementary Data [file 08977151251360617_supplementary_data.docx]

# **SUPPLEMENT**

**Supplement A. Prognostic Risk Score Matching.** To adjust for sex-specific differences in clinical presentation and demographics, prognostic risk scores were calculated (based on logistic regression models comparing female vs. male patients) for each patient including the following parameters (based on the IMPACT score variables): Age, Charlson Comorbidity Index, GCS motor score, pupillary reactivity, Injury Severity Score, presence of isolated TBI, hypotension, hypoxia, and blood glucose. Prognostic risk scores matching was then performed using the nearest-neighbor method with a caliper of 0.2 and 1:1 matching. The results of the matching are shown in A (density distribution of prognostic risk scores before and after adjustment – blue: favourable outcome, red: unfavourable outcome) and B (point distribution of matched and unmatched units). The variables (pre and post matching) are shown in table C below.


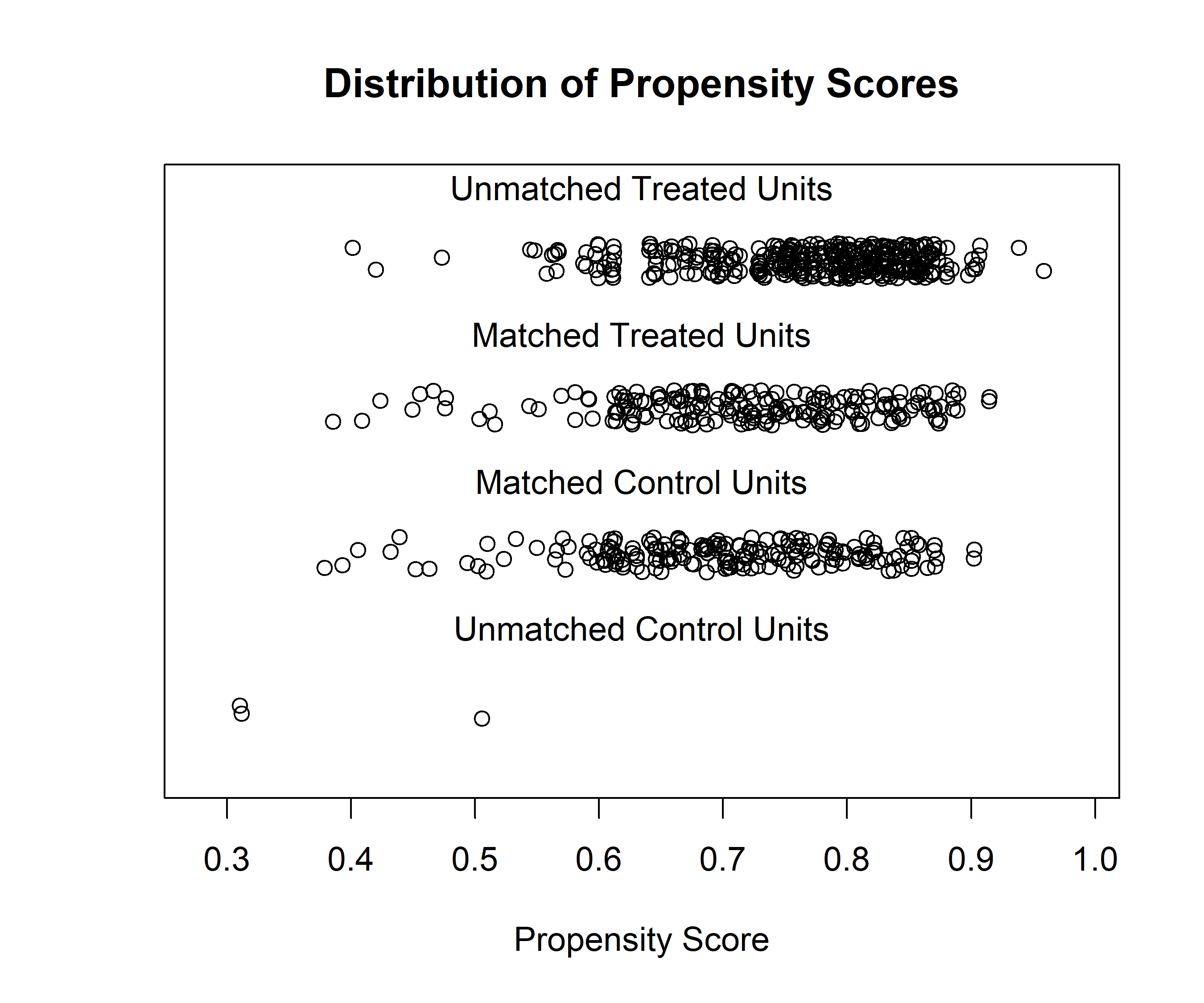

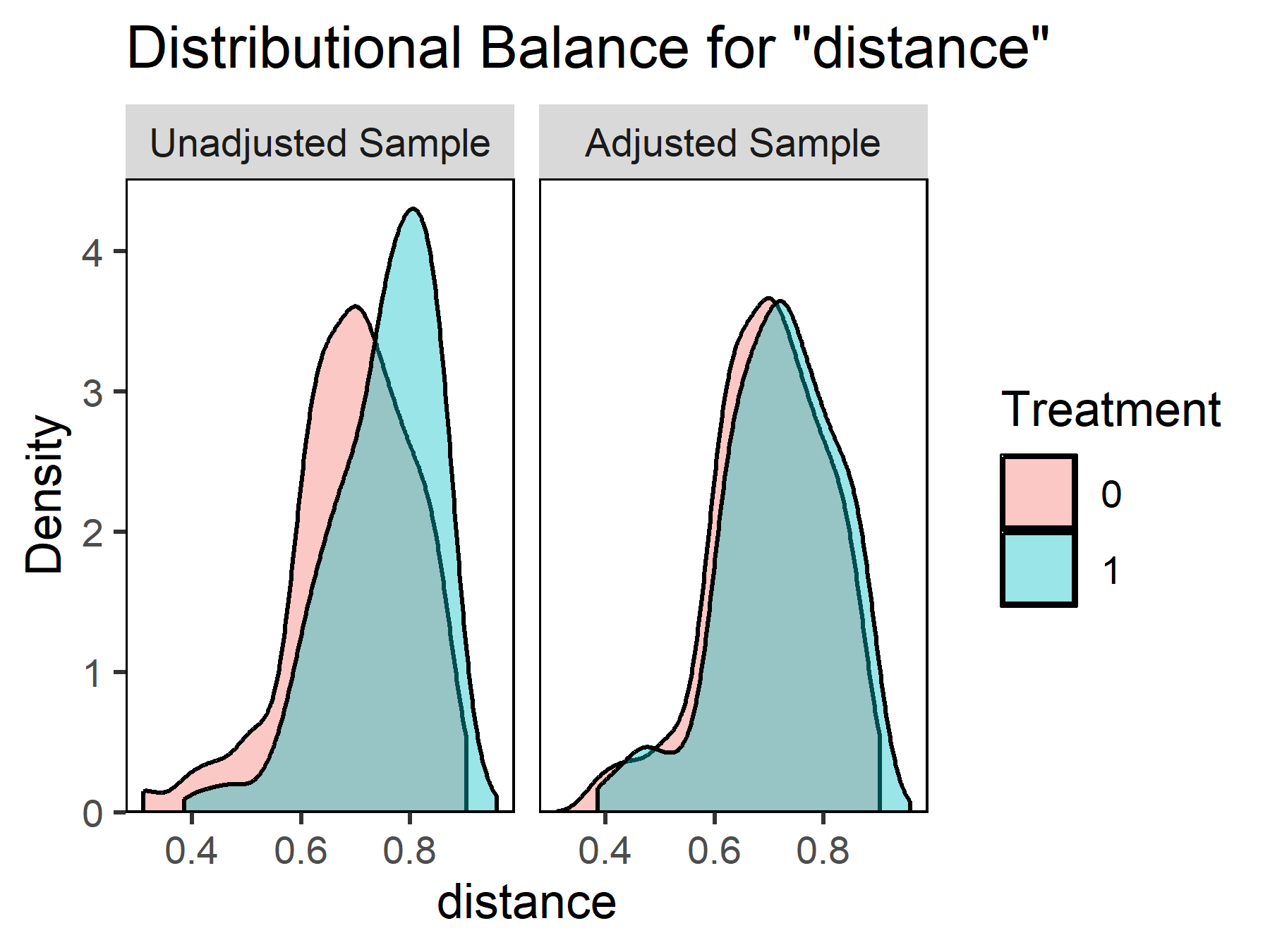


Unmatched: Male

Unmatched: Female

Matched: Female

Matched: Male

**B**

**A**

| **Characteristic** | **female** N = 185 | **male** N = 185 | **p-value** |
| --- | --- | --- | --- |
| Age | 64 (47, 78) | 60 (37, 76) | 0.2 |
| Charlson Comorbidity Index | 0 (0, 1) | 0 (0, 1) | >0.9 |
| GCS M |  |  | 0.4 |
| 1 | 36 (19%) | 36 (19%) |  |
| 2 | 4 (2.2%) | 4 (2.2%) |  |
| 3 | 6 (3.2%) | 3 (1.6%) |  |
| 4 | 20 (11%) | 16 (8.6%) |  |
| 5 | 23 (12%) | 37 (20%) |  |
| 6 | 96 (52%) | 89 (48%) |  |
| Pupillary Reactivity |  |  | 0.6 |
| both reacting | 150 (81%) | 157 (85%) |  |
| None reacting | 29 (16%) | 22 (12%) |  |
| One reacting | 6 (3.2%) | 6 (3.2%) |  |
| ISS | 25 (14, 29) | 22 (16, 29) | 0.6 |
| Isolated TBI | 104 (56%) | 96 (52%) | 0.4 |
| Hypotension | 23 (12%) | 27 (15%) | 0.5 |
| Hypoxia | 59 (32%) | 56 (30%) | 0.7 |
| Blood glucose (mmol/l) | 7.40 (6.20, 8.90) | 7.20 (6.10, 8.50) | 0.2 |

**C**

**Supplement B. Univariable analyses of prognostic risk scores matched subjects.**

**Table B1. Social determinants of health and prior fitness**

| **Characteristic** | **female**, N = 185 | **male**, N = 185 | **p-value** |
| --- | --- | --- | --- |
| Insurance (private) | 21 (11%) | 16 (9%) | 0.4 |
| Civil status |  |  | 0.036 |
| *divorced/separated* | 20 (11%) | 19 (11%) |  |
| *partnership/marriage* | 96 (53%) | 105 (59%) |  |
| *single* | 39 (22%) | 46 (26%) |  |
| *widowed* | 25 (14%) | 9 (5%) |  |
| Living situation |  |  | 0.3 |
| *alone at home* | 53 (32%) | 46 (27%) |  |
| *with other persons at home* | 103 (62%) | 119 (70%) |  |
| *retirement/nursing home* | 10 (6%) | 6 (4%) |  |
| Dependent on Support for ADL (yes) | 28 (15%) | 13 (7%) | 0.014 |
| Income category |  |  | 0.039 |
| *high* | 6 (4%) | 19 (12%) |  |
| *middle* | 126 (81%) | 123 (75%) |  |
| *low* | 23 (14%) | 23 (14%) |  |
| Education |  |  | 0.012 |
| *basic* | 52 (60%) | 75 (63%) |  |
| *higher* | 32 (37%) | 30 (25%) |  |
| *university* | 2 (2%) | 15 (13%) |  |
| Employment status |  |  | 0.094 |
| *employed/working/in training* | 62 (36%) | 82 (46%) |  |
| *retired* | 96 (55%) | 78 (44%) |  |
| *unemployed* | 15 (9%) | 17 (10%) |  |
| Religion |  |  | 0.3 |
| *christian* | 103 (56%) | 90 (49%) |  |
| *religiously unaffiliated* | 45 (24%) | 57 (31%) |  |
| *other* | 37 (20%) | 38 (21%) |  |
| Nationality (Swiss) | 158 (85%) | 154 (83%) | 0.6 |
| Language (German) | 164 (89%) | 146 (89%) | 0.9 |

**Table B2. Social determinants of health: Small Area Variations**

| **Characteristic** | **female**, N = 185 | **male**, N = 185 | **p-value** |
| --- | --- | --- | --- |
| Place of residence (urban) | 142 (77%) | 127 (69%) | 0.08 |
| Proportion welfare dependency | 2.2 (1.7-4.3) | 1.8 (1.7-4.3) | 0.14 |
| Proportion unemployment | 2.4 (1.9-2.4) | 2.2 (1.9-2.4) | 0.044 |
| Median income (in 1000 CHF) | 55.2 (54.2-56.9) | 56.0 (54.2-56.9) | 0.4 |
| Median Assets (in 1000 CHF) | 70 (54-93) | 90 (54-93) | 0.3 |
| Density of private practitioners (per 1000 residents) | 1.7 (0.9-4.7) | 1.8 (0.9-3.9) | 0.6 |
